# Low Complexity Automatic Stationary Wavelet Transform for Elimination of Eye Blinks from EEG

**DOI:** 10.3390/brainsci9120352

**Published:** 2019-12-02

**Authors:** Mohammad Shahbakhti, Maxime Maugeon, Matin Beiramvand, Vaidotas Marozas

**Affiliations:** 1 Faculty of Electrical and Electronics Engineering, Kaunas University of Technology, 51423 Kaunas, Lithuania; vaidotas.marozas@ktu.lt; 2 Biomedical Engineering Institute, Kaunas University of Technology, 51423 Kaunas, Lithuania; 3 UAB Ortho Baltic, 51124 Kaunas, Lithuania; Maxime.Maugeon@orthobaltic.lt; 4 Department of Biomedical Engineering, Dezful Branch, Islamic Azad University, Dezful 6461645169, Iran; matin.beiramvand@gmail.com

**Keywords:** electroencephalography, eye blink, SWT, skewness

## Abstract

The electroencephalogram signal (EEG) often suffers from various artifacts and noises that have physiological and non-physiological origins. Among these artifacts, eye blink, due to its amplitude is considered to have the most influence on EEG analysis. In this paper, a low complexity approach based on Stationary Wavelet Transform (SWT) and skewness is proposed to remove eye blink artifacts from EEG signals. The proposed method is compared against Automatic Wavelet Independent Components Analysis (AWICA) and Enhanced AWICA. Normalized Root Mean Square Error (NRMSE), Peak Signal-to-Noise Ratio (PSNR), and correlation coefficient (ρ) between filtered and pure EEG signals are utilized to quantify artifact removal performance. The proposed approach shows smaller NRMSE, larger PSNR, and larger correlation coefficient values compared to the other methods. Furthermore, the speed of execution of the proposed method is considerably faster than other methods, which makes it more suitable for real-time processing.

## 1. Introduction

Electroencephalographic (EEG) signals recorded from the scalp are extensively used in the medical practice to analyze brain activity for the diagnosis, the management and the investigation of neurological problems such as, but not limited to, epilepsy [1,2], neurodegenerative diseases [3,4] and sleep disorders [5,6]. Another important application for scalp EEG can be found in Brain-Computer Interface (BCI), which has made significant advances in neurorehabilitation and assistive technologies, while also targeting the improvement of quality of life for disabled people [7].

EEG signals are often corrupted by non-cerebral signals originating from physiological and non-physiological sources such as electrooculographic (EOG), electrocardiographic (ECG) and electromyographic (EMG) signals, power line noise, electrode noise, etc. [8]. These sources distort the EEG signal and may affect the final detection or classification results. Artifact removal is, therefore, a critical and necessary step in EEG signal processing.

Amongst physiological artifacts, EOG is considered to have the most impact on the EEG signal analysis, due to its high amplitude and overlapping frequency components. The cornea (positive) and the retina (negative) of the human eye form an electrical dipole. Movements and blinks of the eye modify this dipole and generate an electrical signal known as EOG, inducing strong ocular artifacts in EEG recordings [9]. Eye blinks are characterized by low frequency components (<4 Hz) with a high amplitude which have a symmetrical activity mainly located on the front lobe electrodes (Fp1, Fp2) with low propagation to other EEG channels. Eye movements are also identified as a low frequency signal (<4 Hz) with lower amplitude but higher propagation to other electrode positions [10]. The occurrence of eye blinks is more frequent than eye movements, therefore, the elimination of eye blinks from EEG has gained more attention than the elimination of eye movements.

There are five classes of algorithms that can be used to remove eye blink artifacts from EEG signals: linear filters, parametric filters, blind source separation (BSS) methods, source decomposition methods and combinations of different algorithms.

Linear filtering can be an effective method for the elimination of artifacts from a desired signal if they do not overlap in the same frequency band [11].The linear filter cannot suppress the eye blink artifacts from EEG signals without affecting the underlying cognitive brain process, e.g., language [12] and decision making [13,14]. Therefore, developing methods able to eliminate eye blink contamination from EEG data, especially in those cases where delta band is part of process, are mandatory. However, it should be noted that the linear filter is the most common technique for removing the narrow band artifacts, such as power line noises from biomedical signals.

Adaptive filters [15] are considered to be the most common technique for real time removal of blink artifacts from EEG. They estimate a signal correlated with the blink artifact by using EOG as a reference channel and then filter eye blinks from the recorded EEG. Despite the fast execution, these methods require an extra channel, which increases system complexity for wearable applications, e.g., long-term epilepsy seizure monitoring [16] or mental stress assessment [17].

To overcome the problem of needing an extra channel, Wiener filter [18,19] was proposed for artifact removal from EEG. The objective of this filter is to minimize mean square error (MSE) between the desired signal and its estimation. In order to minimize the MSE, an estimation of the power spectral densities (PSD) of the signal and artifact is performed. Although it does not require an extra channel for EOG recording, initial calibration is necessary. In addition, the filter cannot be applied on-line.

In the absence of the reference artifact channel and prior knowledge of the collected EEG signal, BSS based methods are considered as the most popular and effective techniques for elimination of eye blinks from EEG signals [20]. The basis of those approaches is to find equivalent principal or independent components to the input EEG channels and then perform processing in the transform domain. Principal Component Analysis (PCA) and Independent Component Analysis (ICA) are the most well known algorithms of BSS used for EEG denoising.

Berg et al. [9] investigated PCA for blink artifact elimination from EEG and concluded that its performance is superior to the regression based method. The main drawback of PCA is, orthogonal assumption between EEG and blink artifact, which is often incorrect. Some studies showed PCA fails when the desired EEG signal and blink artifact have the resembling amplitude [21,22]. Many researchers believe that EEG and blink artifact are independent rather than orthogonal [20,23,24], therefore, ICA is applied instead of PCA.

Jung et al. [23] and Vigario [24] used ICA for removal of blink artifacts from EEG signals. One of the drawbacks of ICA is that its effectiveness relies on statistical independence of EEG and blink sources, and thus large amounts of data are required to achieve reliable results [11]. The other drawback is its computational demands [15,18]. However, several studies have proposed the combination of BSS and regression methods for real time denoising of EEG signals, e.g., Guarnieri et al [25] but the recording of the artifact reference was mandatory. Therefore, alternative methods, which do not need an extra channel for the artifact with the possibility of real time processing, are required.

In the last two decades, a wide range of source decomposition methods have been presented for EEG denoising such as Empirical Mode Decomposition (EMD) [26,27], Variational Mode Decomposition (VMD) [28] and Wavelet Transforms (WT) [29,30,31,32].

EMD is an appropriate tool for the analysis of nonlinear and nonstationary signals and has gained attention for biomedical signal analysis. It decomposes the signal into a series of basis functions called Intrinsic Mode Functions (IMF), and one residue. Little robustness to noise is the major limitation of EMD as its performance could be affected by white noise. To solve this problem, Ensemble EMD (EEMD) [33] and VMD [28] algorithms were proposed.

The principle of the VMD is to decompose a signal into several Band Limited Intrinsic Mode Functions (BLIMFs). Biswal et al. [28] applied VMD algorithm to remove blink artifacts from frontal EEG electrodes and compared the performance of the proposed method to EMD and EEMD. VMD outperformed the methods under comparison. The sensitivity of EMD to white noise was resolved by VMD but the computational complexity was increased significantly.

WT has been applied for EEG signal denoising. The transformation is expressed as an inner product of the signal with the time scaled and shifted versions of the wavelet (basis) function. It decomposes the signal into sets of detail (high frequency) and approximation (low frequency) coefficients. Among different WT algorithms, discrete wavelet transform (DWT) is used frequently for artifact reduction in biomedical signals [34,35]. Nonetheless, stationary wavelet transform (SWT) performed better for EOG reduction in EEG as it provided better temporal resolution [36]. Effective separation of the desired signal and the artifact components with SWT relies on the correct determination of mother wavelet (basis function) and the level of decomposition.

Even though WT based methods are quite popular for EEG processing, the combinations of WT and BSS techniques have also gained a lot of attention for EEG artifact reduction in recent literature [37,38,39,40]. The most prominent example of these methods could be wavelet ICA (WICA) algorithm, which was initially introduced by Azzerboni et al. [37] to remove ECG artifacts from surface EMG. However, WICA was not automatic, as the selection of the wavelet components for ICA processing was performed manually. Castellanos et al. [38] proposed wavelet enhanced ICA to detect and eliminate EOG and ECG artifacts from EEG but only single EOG and ECG artifact waveforms were used to evaluate the performance. Two automatic WICA based methods: Automatic Wavelet Independent Components Analysis (AWICA) and Enhanced AWICA (EAWICA) have been described in [39,40] to remove electrical shift, linear trend, temporal muscle, and eye blinks from EEG signals. However, the performance of AWICA and EAWICA techniques is sensitive to five parameters set before processing. Furthermore, those methods are computationally expensive due to the implementation of ICA.

The aim of this research is to propose an automatic low complexity algorithm based on SWT for the filtering of eye blink contaminated EEG signals. The method requires only two parameters to be specified before processing, mother wavelet and level of decomposition. Selection of mother wavelet is based on the similarity of the desired signal and the mother wavelet; thus, determination of the level of decomposition plays the most important role in automatic SWT (ASWT) denoising method. In this paper, we propose to use the difference in skewness between two consecutive approximation coefficients levels to stop the decomposition process of SWT automatically when it reaches the eye blink artifact components.

The remainder of this paper is organized as follows: Section 2 describes and explains the proposed method and performance evaluation, Section 3 describes the data, and results are presented in Section 4. Finally, further discussion on the achieved results and concluding notes are given in Section 5 and Section 6.

## 2. Artifact Removal Methods

### 2.1. Proposed ASWT Method

SWT passes a signal through high-pass and low-pass filters to decompose it into high and low frequency components, called detail and approximation coefficients respectively. The main advantage of SWT is overcoming translation-invariance of DWT which is achieved by removing downsamplers and upsamplers. As a result, the coefficients of SWT contain the same number of the samples as the original signal.

Two parameters are required to be determined before conventional SWT processing: the mother wavelet and the number of decomposition levels. The selection of the mother wavelet is generally based on similarities between the mother wavelet and the desired signal. Daubechies wavelet ‘db4’ is commonly used as the mother wavelet for biomedical signal processing [37,38,39,40] as its morphology resembles eye blink signal. The spectrum of eye blink artifact varies between 0.01 to 3 Hz [10,38,39,40]. Therefore, it is expected to increase the power of the frequencies in the lower end of the EEG spectrum, and as a result, the eye blink components lie in the last several levels of the SWT.

**Figure 1:**
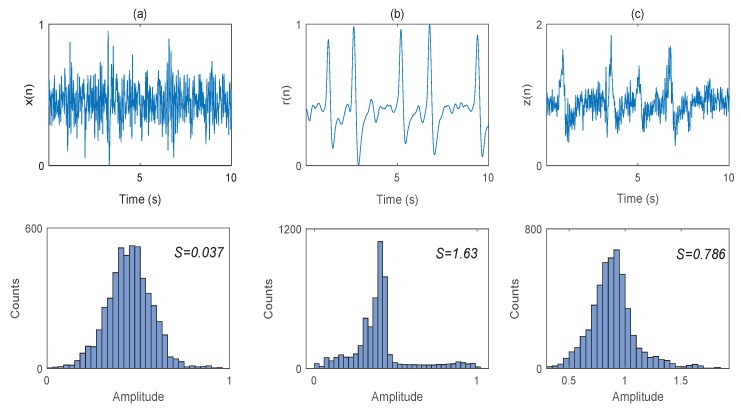
Examples of signals and histograms for: clean EEG (**a**), blink artifact (**b**) and contaminated EEG (**c**). *S* is the skewness value.

One of the goals of this paper is to introduce a criterion for stopping the SWT decomposition when it reaches the eye blink components. Since blinking is a low frequency phenomenon, it is expected to appear in approximation coefficients of SWT. Hence, the proposed criterion should be enforced on those coefficients. The presence of eye blinks may be indicated by a higher absolute value of skewness as it has a considerably larger amplitude compared to uncontaminated EEG signal [41,42]. Figure 1 illustrates the examples of the signals and their histograms for pure EEG, blink artifact and blink contaminated EEG signal.

Skewness, *S*, for a sample of N values of the signal ’x’ is defined as follows [43]:

(1) $$S=\frac{\frac{1}{N}\sum_{i=1}^{N}{\left(x_{i}-\bar{x}\right)}^{3}}{{\sqrt{\frac{1}{N-1}\sum_{i=1}^{N}{\left(x_{i}-\bar{x}\right)}^{3}}}^{2}},$$

where $\bar{x}$ is the mean of the sample.

**Figure 2:**
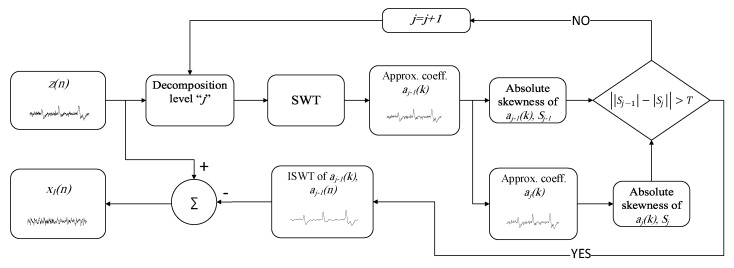
The block diagram of the proposed method.

For automatic stoppage of SWT when it reaches the blink artifact components, we have applied a criterion based on the absolute difference of the absolute skewness values of two consecutive approximation coefficients levels. The block diagram of the proposed algorithm is shown in Figure 2.

The proposed criterion is defined as absolute difference of absolute skewness values of two consecutive approximation coefficients in SWT domain, which is expressed as follows:

(2) $$\delta =||S_{j}\left|-\right|S_{j-1}||,$$

where *S* is the skewness and *j* is the level of decomposition of SWT. If δ>T, we can assume that SWT has reached the blink components. The approach to extract and remove eye blink components from the contaminated EEG is presented as follows:

1. Apply *j* level SWT to EEG signal contaminated with eye blinks, z(n), and extract the approximation $a_{j-1}\left(k\right)$ and $a_{j}\left(k\right)$ coefficients, where j=2,3,…,J (wavelet domain).
2. Compute the absolute difference of absolute skewness values of $a_{j-1}\left(k\right)$ and $a_{j}\left(k\right)$ as δ.
3. If δ>*T*, inverse SWT (ISWT) of $a_{j-1}\left(k\right)$ in order to get $a_{j-1}\left(n\right)$ which is considered as the eye blink artifact. Subtract $a_{j-1}\left(n\right)$ from the contaminated EEG signal z(n) to obtain the filtered EEG signal. Otherwise, go back to step 1 and proceed to j=j+1.

In this paper, six *T* values ranging from 0.05 to 0.3 with a step of 0.05 have been applied. The optimal value of *T* was selected based on lowest mean ± std of error between pure and filtered EEG signals.

### 2.2. Methods Under Comparison

The performance of the proposed algorithm is compared with AWICA and EAWICA algorithms which are available from the authors upon request. There are five parameters required to be set before processing for those algorithms. Optimal settings were set as described in [40].

### 2.3. Performance Evaluation

Normalized root mean square error (NRMSE), peak signal-to-noise ratio (PSNR) and correlation coefficient (ρ) between pure and filtered EEG signals are the principal performance measures in time domain. NRMSE and PSNR evaluate the magnitude distortion and ρ investigates phase distortion of the filtered EEG signals, respectively. NRMSE is defined as follows:

(3) $$NRMSE=\frac{\sqrt{\frac{1}{N}\sum_{i=1}^{N}{\left(x\left(n\right)-x_{1}\left(n\right)\right)}^{2}}}{max_{x(n)}-min_{x(n)}}\times 100,$$

where x(n) is pure EEG signal and $x_{1}\left(n\right)$ is filtered EEG signal.

PSNR is the peak error measurement which is expressed in decibels [40]:

(4) $$PSNR=20*\log_{10}\left(\frac{max_{x(n)}}{\sqrt{\frac{1}{N}\sum_{i=1}^{N}{\left(x\left(n\right)-x_{1}\left(n\right)\right)}^{2}}}\right).$$

Correlation coefficient is a value between 0 to 1 which is expressed as:

(5) $$\rho =\frac{cov(x\left(n\right),x_{1}\left(n\right))}{\sigma_{x}\left(n\right)\sigma_{x_{1}}\left(n\right)},$$

where cov is covariance and σ is variance.

The algorithms have been executed on a computer with 3.2 GHz core i7 CPU, 16 GB RAM and with widely used computing software MATLAB R2018a (Mathworks Inc., Natick, MA, USA).

## 3. EEG Data Description

### 3.1. Simulated EEG signals

**Figure 3:**
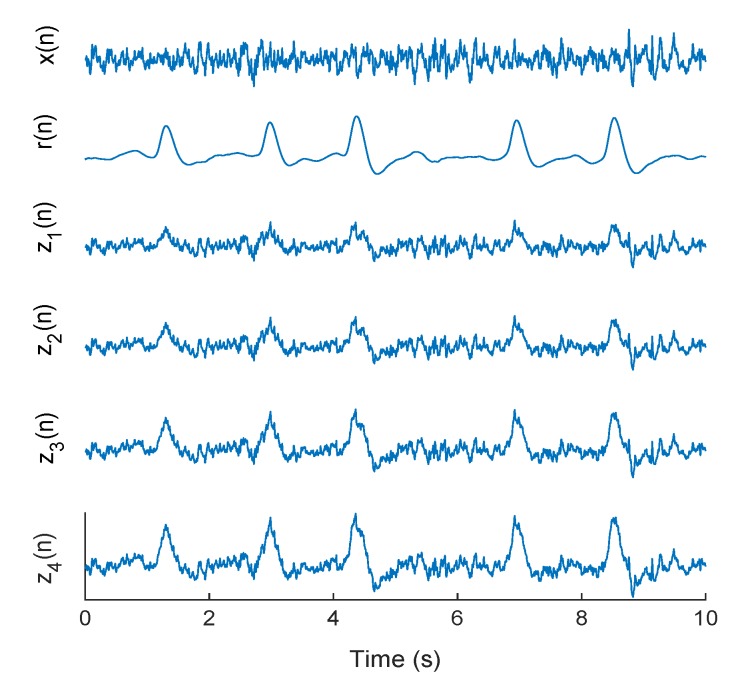
Examples of simulated data from CHB-MIT database: x(n)—pure EEG, r(n)—eye blink artifact, $z_{1}\left(n\right)$—contaminated EEG with a=0.75, $z_{2}\left(n\right)$—a=1.0, $z_{3}\left(n\right)$—a=1.5, and $z_{4}\left(n\right)$—a=2.0.

Twenty four EEG signals from the CHB-MIT Scalp EEG database [44,45] (sampling rate Fs=256 Hz) have been selected to develop the proposed algorithm. We manually cut out 10 s long artifact-free EEG epochs, and in this way, we were able to collect pure EEG signals. 30 EEG signals collected during mental arithmetic tasks (EEG-MAT) (Fs=500 Hz) [44,46] have been selected to test the performance of the proposed algorithm. EEG-MAT database had already been filtered and the data was clean. To generate simulated EEG signals contaminated with eye blinks, 54 real eyeblink signals from the BCI experiment for motor imagery movement of the left and right hand [47], and the BCI Competition 2008 (Graz Data Sets 2a) [48] have been used. The eye blink signals were band-pass filtered between 0.1 and 3 Hz and resampled to 256 and 500 Hz. Simulated EEG signals contaminated with eye blinks were then generated by an additive model of clean EEG and blink signals. The additive model is described as follows:

(6) z(n)=x(n)+ar(n),

where z(n) is the EEG signal contaminated with eye blink artifact, x(n) is the pure EEG and r(n) is the eye blink artifact. Since propagation of blink artifact is not equal in different EEG electrodes, the term ’*a*’ with four different values is applied to put emphasis on this fact that eye blink magnitude is not equally distributed for all EEG electrodes [49]. Therefore, the developing set of the algorithm includes a total of 24 × 4 = 96 and test set contains 30 × 4 = 120 of simulated signals. Figure 3 demonstrates examples of pure EEG, eye blink artifact, and blink contaminated EEG signals with different values of *a* for CHB-MIT database.

### 3.2. Real EEG Signals

In order to assess the performance of the proposed method on real EEG signals contaminated with eye blinks, 8 EEG signals from the BCI experiment for motor imagery movement of the left and right hand (fs=512 Hz) [47] and 8 EEG signals from the BCI Competition 2008 (Graz Data Sets 2a) (fs=250 Hz) [48] with length of 60 s have been used. Each signal was divided into 6 windows of 10 s and then the denoising methods were applied. An eye blink reference channel was recorded simultaneously to EEG data for both databases. All raw EEG signals were band pass filtered between 0.01 to 40 Hz and then the algorithms were performed to denoise the EEG signals.

## 4. Results

**Figure 4:**
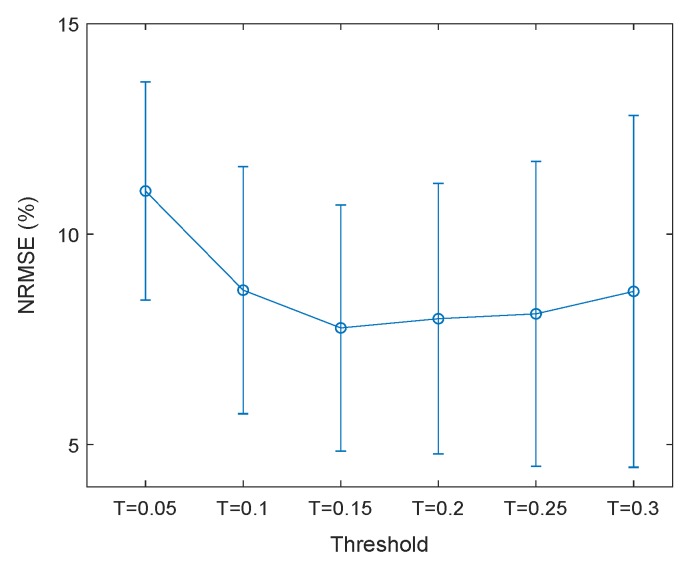
Mean ± std of NRMSEs per different *T* values.

Figure 4 shows the mean ± std of NRMSE between pure and filtered EEG signals per different values of *T* for CHB-MIT database. As observed below, the *T* value of 0.15, has the lowest NRMSE.

**Figure 5:**
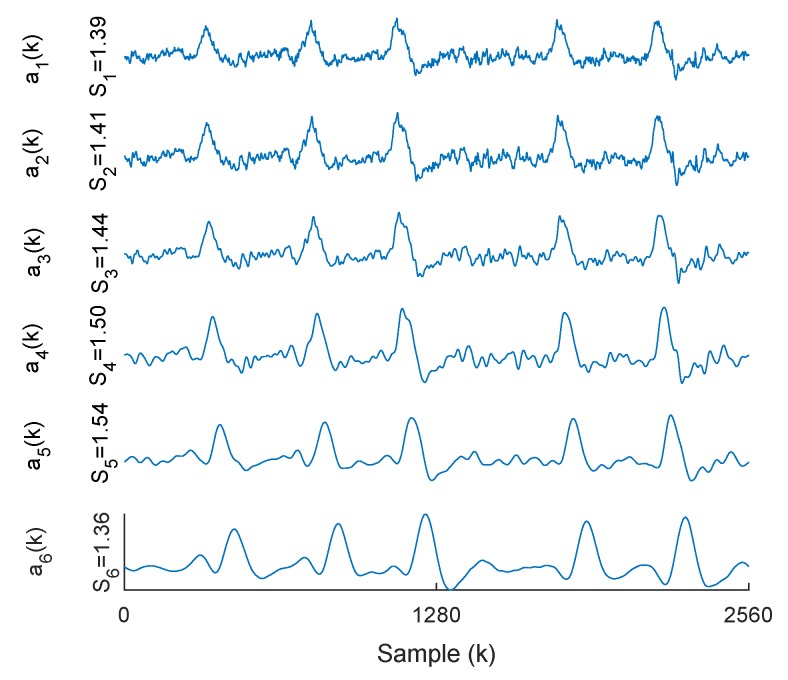
Examples of approximation coefficients and corresponding skewness values for a contaminated EEG signal from CHB-MIT database in SWT domain.

Figure 5 shows an example of a decomposed contaminated EEG signal into approximation coefficients from CHB-MIT database in SWT domain. According to the proposed method, inverse SWT of $a_{5}\left(k\right)$ is considered as the eye blink artifact and is removed from the contaminated EEG.

**Figure 6:**
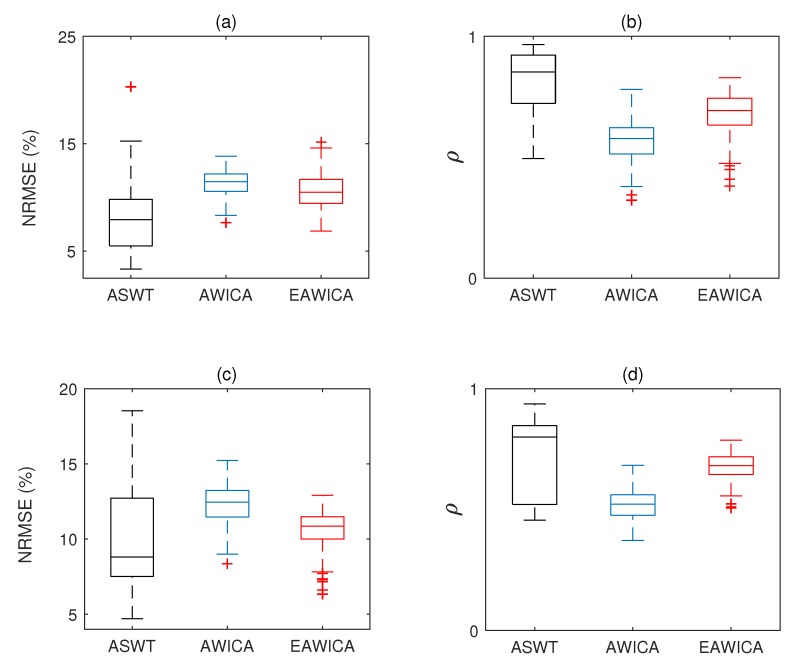
Box plots of NRMSE and correlation coefficient between pure and filtered EEG for simulated data by all methods: (**a**,**b**) are for CHB-MIT, (**c**,**d**) are for EEG-MAT databases.

Quantitative analysis, computation of NRMSE, PSNR and correlation coefficient between pure and filtered EEG signals were solely performed for simulated data. For real EEG signals contaminated with eye blinks, performances are evaluated visually. Figure 6 illustrates the box plots of NRMSE and correlation coefficient values between pure and filtered EEG signals for different databases and algorithms.

**Figure 7:**
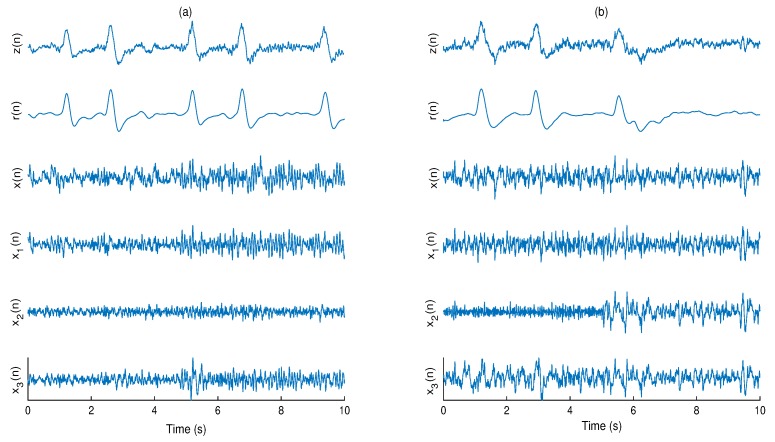
Examples of eye blink cancellation in simulated EEG signals from: EEG-MAT (**a**), and CHB-MIT (**b**) databases: *z*(*n*)—contaminated EEG, *r*(*n*)—real eye blink artifact, *x*(*n*)—pure EEG, $x_{1}\left(n\right)$—filtered EEG by the proposed method, $x_{2}\left(n\right)$—filtered EEG by the AWICA and $x_{3}\left(n\right)$—filtered EEG by the EAWICA.

Figure 7 shows examples of the contaminated, pure, and filtered EEG signals from both databases with all methods.

**Figure 8:**
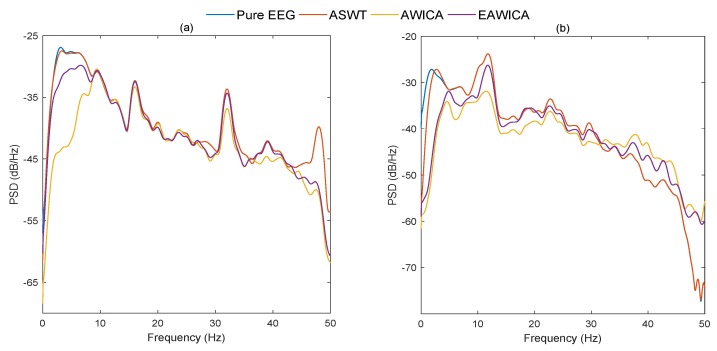
Examples of the PSDs for the pure and the filtered EEG signals by all methods for simulated data using CHB-MIT (**a**) and EEG-MAT (**b**) databases.

An example of Power Spectral Density (PSD) for the pure and the filtered EEG signals from both databases are shown in Figure 8. As it may be observed, ASWT could preserve EEG components better than methods under comparison.

**Figure 9:**
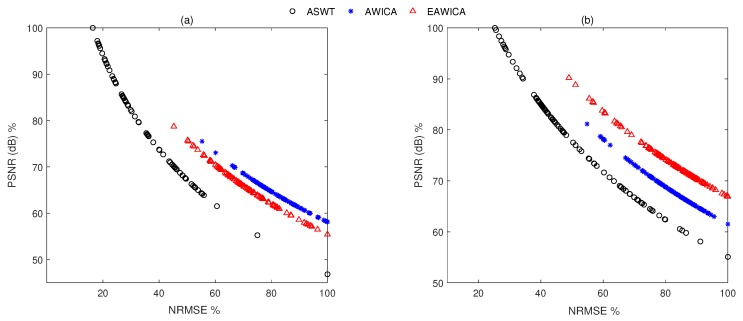
PSNR curves as the function of NRMSE for filtered EEG signals: CHB-MIT (**a**) and EEG-MAT (**b**) databases. ASWT outperformed the other algorithms because in each subplot, the points associated with the largest PSNR and the smallest NRMSE were achieved by ASWT.

Figure 9 shows PSNR curves as the function of NRMSE for the filtered EEG signals using all methods. As it can be seen, the proposed method gives higher PSNR and lower NRMSE for most of signals than methods under comparison.

**Figure 10:**
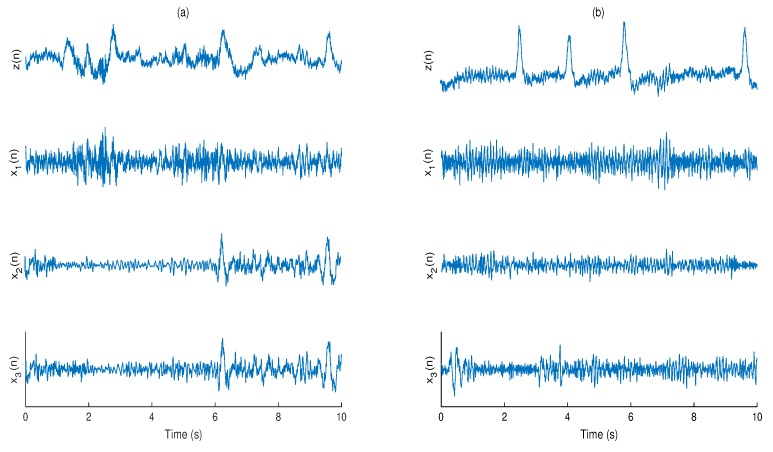
Examples of eye blink cancellation in real EEG signals from: BCI Competition 2008—Graz Data Sets 2a (**a**), and BCI 2011 left/right motor imagery (**b**). *z*(*n*)—EEG contaminated with eye blink, $x_{1}\left(n\right)$—filtered EEG by the proposed method, $x_{2}\left(n\right)$—filtered EEG by AWICA and $x_{3}\left(n\right)$—filtered EEG by EAWICA.

Ten seconds fragments of the real and filtered EEG signals resulting from all methods are illustrated in Figure 10. Considering that both EEGs and eye blinks were unknown in the original EEG signals, NRMSE, PSNR, and correlation coefficient are not regarded as evaluation criteria of the artifact removal, therefore we have to confine ourselves to visual assessment.

Computational time for the implementation of the algorithm is of great importance for on-line applications. Table 1 shows the mean ± std of the computational time expressed in seconds for different algorithms and databases per 20 times run.

## 5. Discussion

Many approaches for eye blink artifact removal from EEG signals have been already described in literature. The main drawbacks of such methods are the necessity of an extra eye blink channel recording for those based on adaptive filter, the lack of performance of linear filtering when the target signal and artifacts overlap in the same frequency band, the computational expensiveness of BSS methods, and the manual setting of the level of decomposition for source decomposition methods.

In this paper, we described an automatic low complexity algorithm called ASWT method to remove eye blink artifacts from EEG signal. The innovation of the method presented in this paper resides in combining SWT based decomposition with skewness analysis for automatic selection of a final wavelet decomposition level to extract and subtract eye blink components from the EEG signals. The main assumption for selection of skewness based criteria is a pronounced asymmetry of amplitude values of the EEG signal at the eye blinks dominated episodes.

The performance and implementation of the proposed method was compared against AWICA and EAWICA algorithms [39,40] which were proposed for automatic artifact reduction from EEG signals. The correlation coefficient, PSNR, and NRMSE have been computed as the criteria to evaluate the performance of the proposed algorithms. The largest PSNR, and correlation coefficient, and the smallest NRMSE, yield the best performance and are interpreted as optimum.

In total, 216 simulated eye blink contaminated EEG signals from two databases have been processed. Figure 6 shows the box plots of the correlation coefficient and NRMSE between pure and filtered EEG signals of both simulated databases for all algorithms. It can be seen, in most of the cases, ASWT outperforms the other algorithms. Indeed, in 52 out 216 simulated signals AWICA and EAWICA performed better than ASWT. Examples of visual evaluation of eye blink filtering by all algorithms and corresponding pure EEG signals for both databases are shown in Figure 7. Figure 8 shows the examples of the corresponding PSD for the pure and the filtered EEG signals for both simulated databases. It could be observed that lesser attenuation and distortion of the filtered EEG was achieved by ASWT. Moreover, PSNR curve as the function of NRMSE for both databases are shown in Figure 9 (each PSNR was normalized with regard to the highest PSNR value and multiplied by 100) [40]. It is clear that ASWT performed superior for the majority of the simulated data. In order to investigate the performance of the proposed method for real life EEG signals, 16 real eye blink contaminated EEG signals from two databases have been used. Figure 10 demonstrates the examples of the real noisy and filtered EEG signals for both databases. It might be seen that the proposed method could eliminate eye blinks with lower distortion of EEG signals.

Computational complexity is another important factor for the usability of the artifact removal approaches. The comparison of algorithms execution for different databases is shown in Table 1. For 96 simulated signals from CHB-MIT database with Fs=256 Hz the proposed method was 12× faster than AWICA and 10× faster than EAWICA. As the sampling frequency increases, the algorithms require a longer time to be executed. ASWT was 27× and 25× faster than AWICA and EAWICA, respectively, for 120 simulated signals from EEG-MAT database with Fs=500 Hz. Additionally, as the length of signals increased for real eye blinks contaminated EEG signals, execution of the AWICA and EAWICA required a considerably longer time than ASWT. The computational time needed for the algorithms under comparison was considerably higher than for the proposed method, thus, they can not be applied for real-time processing.

AWICA and EAWICA methods require setting of five parameters before processing and consequently, the performance of the algorithms relies on the accurate setting of these parameters. More user-independency is the main advantage of the proposed method as only one parameter is required to be set before denoising.

The major limitation of the current experiment is the absence of other sources of artifacts in eye blink contaminated EEG signals, while in real life, EEG is contaminated with different artifacts simultaneously. Future work will be devoted to the improvement of the algorithm (i) to detect the eye blink contaminated epochs and filter only contaminated epochs, (ii) to account for other types of artifacts EMG, ECG, and electrode noise.

## 6. Conclusions

Eye blinks are the most common artifacts distorting EEG signals. This research proposed a low complexity approach based on stationary wavelet transform and skewness to suppress eye blink artifacts from EEG signals. The performance of the proposed algorithm was compared with AWICA and EAWICA methods by using four databases of EEG signals. Obtained results highlighted (i) better or comparable denoising performance and (ii) faster execution of the proposed method. More independence from the user is another advantage of the proposed method. AWICA and EAWICA algorithms require five parameters to be specified before processing, whereas ASWT requires only one parameter.

## Figures and Tables

**Table 1:** Mean ± STD of computational time for the implementation of the algorithms, expressed in seconds.

Method	AWICA	EAWICA	ASWT
**Database**			
CHB-MIT	22.8 ± 4.5 s	18.5 ± 2.3 s	1.9 ± 0.24 s
EEG-MAT	78.8 ± 5.8 s	71.3 ± 6.3 s	2.8 ± 0.67 s
BCI Competition	46.8 ± 4.2 s	44.3 ± 5.2 s	1.8 ± 0.34 s
BCI motor imagery	105.3 ± 8.7 s	84.5 ± 6.4 s	2.7 ± 0.62 s
